# Cardiometabolic comorbidities in obstructive sleep apnea patients are related to disease severity, nocturnal hypoxemia, and decreased sleep quality

**DOI:** 10.1186/s12931-020-1284-7

**Published:** 2020-01-29

**Authors:** Stephanie André, Fabio Andreozzi, Chloé Van Overstraeten, Sidali Ben Youssef, Ionela Bold, Sarah Carlier, Alexia Gruwez, Anne-Violette Bruyneel, Marie Bruyneel

**Affiliations:** 1 Department of Pulmonary Medicine, Saint-Pierre University Hospital, Rue Haute 322, 1000 Brussels, Belgium; 2 Université Libre de Bruxelles, Brussels, Belgium; 3 Department of Pulmonary Medicine, Brugmann University Hospital, Brussels, Belgium; 4 KiCarre, research department, Lentilly, France

**Keywords:** Diabetes, Cardiovascular comorbidities, Dyslipidemia, Obstructive sleep apnea, Nocturnal hypoxemia, Sleep quality, Polysomnography

## Abstract

**Background:**

Obstructive sleep apnea syndrome (OSA) is currently recognized as an independent risk factor for hypertension, arrhythmia, coronary heart disease, stroke, and metabolic disorders (e.g. diabetes, dyslipidemia). In clinical practice, apnea-hypopnea index (AHI) is the marker used to classify disease severity and guide treatment. However, AHI alone does not sufficiently identify OSA patients at risk for cardiometabolic comorbidities. With this in mind, the aim of this retrospective study was to determine whether some polysomnographic parameters (e.g. apnea-hypopnea duration, sleep structure, nocturnal hypoxemia) are specifically associated with cardiometabolic comorbidities in OSA.

**Methods:**

In this retrospective study, 1717 patients suffering from moderate/severe OSA were included between 2013 and 2017. Data on demographics, comorbidities, and polysomnographic characteristics were collected and analyzed to identify factors associated with cardiometabolic complications.

**Results:**

The medical files of 1717 patients (68% male) were reviewed. The mean AHI was 43.1 +/− 27.7 with 57.3% of patients suffering from severe OSA, and 52% from at least one cardiovascular comorbidity (CVCo). Diabetes affected 22% of the patients and 27% exhibited dyslipidemia. Patients affected by CVCos were older, and more often women and non-smokers. These patients also had worse sleep quality, and a more marked intermittent/global nocturnal hypoxemia. With regard to diabetes, diabetics were older, more often non-smoker, non-drinker women, and were more obese. These patients also exhibited more severe OSA, especially in non-REM (NREM) sleep, worse sleep quality, and a more marked intermittent/global nocturnal hypoxemia. Dyslipidemia was more frequent in the absence of alcohol consumption, and was associated with OSA severity, decreased sleep quality, and longer AH in REM sleep.

**Conclusions:**

This study identifies demographic and polysomnographic factors associated with cardiometabolic comorbidities. Patients (especially women) suffering from more severe OSA, longer sleep apneas and hypopneas, worse sleep quality, and marked intermittent/global nocturnal hypoxemia are more likely to develop cardiometabolic comorbidities. This should stimulate clinicians to obtain adequate treatment in this population.

## Background

Obstructive sleep apnea (OSA) syndrome is a common medical problem. The prevalence of the disorder is increasing and is related to its main causal factors, namely obesity and aging [1]. OSA is now recognized as an independent risk factor for hypertension, arrhythmia, coronary heart disease, and stroke [2–4]. Metabolic disorders (diabetes, impaired lipid metabolism) are also associated with OSA [5, 6].

Despite these associations, the clinical picture of OSA is not homogeneous and can differ widely among patients. In the last decade, a variety of clinical phenotypes have been identified, highlighting clusters with different symptomologies and morbidities in patients affected by the “same” OSA [7]. Response to treatment is also impacted by these clinical phenotypes. For example, Gagnadoux et al. showed that clusters defined as “female OSA”, “mildly symptomatic OSA”, and “comorbid OSA” were less likely to use continuous positive airway pressure (CPAP) successfully [8].

In clinical practice, apnea-hypopnea index (AHI) is still the marker used to classify disease severity and guide treatment, but it is a limited marker that doesn’t sufficiently describe the clinical picture of OSA. For example, among patients with the same AHI, some exhibit long apneas with profound oxygen desaturation, and others very short apneas without significant associated hypoxia. These differences can potentially lead to several clinical OSA phenotypes that should maybe not be managed similarly.

With this in mind, the aim of this retrospective study was to identify whether some particular polysomnographic parameters (e.g. apnea-hypopnea duration, sleep structure, nocturnal hypoxemia) are specifically associated with cardiometabolic comorbidities in OSA patients.

## Methods

This study was performed in the sleep unit of the Saint-Pierre University Hospital in Brussels, Belgium (tertiary referral center).

### Study design

The study was retrospective, based on prospectively collected data in our sleep lab. All naïve patients suffering from moderate-to-severe sleep apnea syndrome (apnea-hypopnea index (AHI) ≥ 15/h of sleep) diagnosed by one single attended polysomnography (PSG) between 01/01/2013 and 12/31/2017 were included.

Data for medical history, treatments, and polysomnographic characteristics were extracted from patient medical files.

Polysomnographies were all performed with the same polysomnographic device in the sleep laboratory of CHU St. Pierre (Brainnet3 IP, Medatec, Belgium).

### Data collection

Age, sex, body mass index (BMI), neck circumference (NC), and alcohol and tobacco use were collected.

Medical files were analyzed in order to collect data on comorbidities (e.g. arterial hypertension (HTA), diabetes, atrial fibrillation). Congestive heart failure was defined by a left ventricular ejection fraction below 35%. PSG was scored according to American Academy of Sleep Medicine 2012 scoring rules [9] and reviewed in order to collect, for all patients, AHI (global, supine/non supine, rapid eye movement (REM) sleep), mean sleep apnea/hypopnea (AH) duration (according to sleep stage), total sleep time (TST), sleep efficiency (SE), sleep stage proportions, periodic leg movements (PLM), oxygen desaturation index (ODI), total duration of oxygen desaturation < 90%, arousal index (ARI), minimal oxygen (0_2_) saturation, and mean nocturnal heart rhythm (HR).

Supine predominant OSA was considered when AHI supine was ≥ two-fold non-supine [10], and REM Predominant when AHI in REM sleep was ≥ two-fold more than in non-REM sleep [11].

The study protocol was approved by the Saint-Pierre University Hospital ethics committee (CE/18–01-06).

### Statistical analysis

Statistical analyses include descriptive statistics. Characteristics of the study population are presented as means with standard deviations for quantitative data and as percentages for qualitative data for the entire cohort and sub-groups. We compared data according to the presence of diabetes (with/without diabetes), cardiovascular comorbidities (with/without), and dyslipidemia (with/without).

In order to compare baseline parameters between groups, an Analysis of variance (ANOVA) with Newman Keuls post-hoc test for quantitative variables (BMI, neck circumference, alcohol, and PSG parameters) or chi-square tests for qualitative variables (sex, ethnicity, smoking, supine, and REM predominant) were applied to compare demographic and polysomnographic data.

To explore possible factors that were associated with diabetes, cardiovascular comorbidities, and dyslipidemia, logistic regression analysis or multiple regression analysis were also used. The regression was adjusted by age, BMI, and smoking status for diabetes, by age, BMI, smoking, sex, and dyslipidemia for cardiac comorbidities, and by age, sex, and BMI for dyslipidemia.

A *p* value below 0.05 was considered as statistically significant. All analyses were performed using Statistica software (v. 6, StatsoftTM) and SPSS version 22.0 (SPSS Inc., Chicago, IL, USA).

## Results

### Demographics

The study included 1717 patients, 1144 males and 573 females. The mean age for the whole population was 52.8 ± 12.7 years old. Females were slightly older than males (females: 54.35 ± 12.76 y vs. males: 52.08 ± 12.62 y). A total of 68% of patients were obese. Patient characteristics are summarized in Table 1.

**Table 1:** Demographic characteristics of the patients

	Study Group *N* = 1717
Age (years) Mean + SD	54.14 ± 12.76
Sex (%)	Female: 32.46
	Male: 67.54
BMI (kg/m^2^) Mean + SD	34.29 ± 8.42
NC (cm) Mean + SD	42.81 ± 4.84
Smokers (%)	Current smokers: 25.40
	Non-smokers: 74.60
	Former smokers: 4.61
Alcohol (%)	Non-drinkers: 39.79
	Drinkers: 60.21
	StD:24.18
	LD:4.80
Unit/day Mean + SD	0.69 ± 1.23

*SD* Standard deviation, *BMI* Body mass index, *NC* Neck circumference, *StD* Strong drinkers (>3 unit/day), *LD* Lower drinkers

### Polysomnography

The mean apnea-hypopnea index (AHI) was 43.1 ± 25.7 with 57.30% of patients suffering from severe OSA (AHI tot >30).

### Comorbidities

**Fig. 1:**
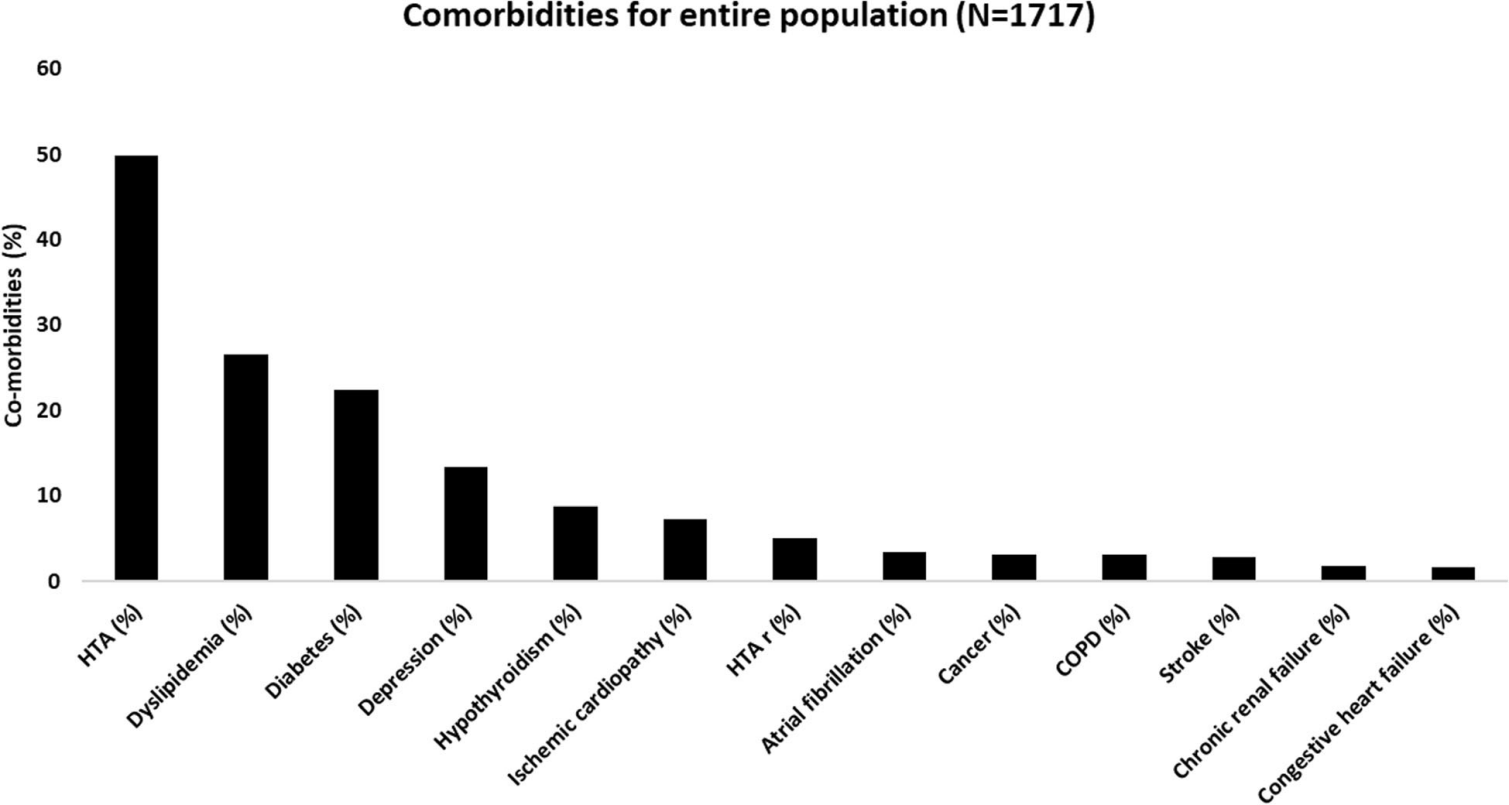
Comorbidities among included patients. COPD: chronic obstructive pulmonary disease, HTA: hypertension, HTA r: resistant hypertension

Fifty-two percent of patients suffered from at least one cardiovascular comorbidity (HTA, atrial fibrillation, ischemic cardiomyopathy, congestive heart failure (left ventricular ejection fraction <35%), or cerebral stroke). Metabolic comorbidities were also frequent, as diabetes affected 22% of patients and dyslipidemia 27% (Fig. 1).

Patients received pharmacological treatment accordingly but compliance to prescribed medications remained unknown.

### Diabetes

Comparing diabetics and non-diabetics, diabetics were older, more often non-smoker non-drinker women, but more obese. They also exhibited more severe OSA, especially in non-REM (NREM) sleep, worse sleep quality (reflected by low SE, TST, percentage REM sleep on TST), more marked intermittent (reflected by ODI) and global (reflected by mean oxygen saturation, oxygen desaturation, and minimal oxygen saturation) nocturnal hypoxemia and a higher mean heart rhythm (HR) (Tables 2 and 3).

**Table 2:** Demographic characteristics for all cardiometabolic comorbidities

	Diabetes *n* = 386	No diabetes *n* = 1331	*p* value	CVCo *n* = 671	No CVCo *n* = 1046	*p* value	Dyslipidemia *n* = 453	No dyslipidemia *n* = 1253	*p* value
Age (mean ± SD)	58.33 ± 11.41	51.23 ± 12.64	0.0001	57.63 ± 11.42	47.65 ± 11.98	<0.0001	58.20 ± 11.49	50.84 ± 12.56	<0.0001
Sex (%)	F: 37.31 M: 62.69	F: 32.30 M: 67.70	0.0023	F: 37.74 M: 62.26	F: 28.64 M: 71.36	0.0001	F: 33,33% H: 66.66%	F: 33,44% H: 66.56%	NS
BMI (kg/m2)	35.52 ± 7.48	33.70 ± 8.14	0.0001	34.73 ± 7.59	33.46 ± 8.41	NS	34.36 ± 7.46	34.02 ± 8.21	NS
NC (cm)	43.22 ± 4.68	42.32 ± 4.74	0.0011	42.75 ± 4.56	42.30 ± 4.92	NS	43.03 ± 4.70	42.34 ± 4.73	0.0181
Smoking (%)	S: 15.80 NS: 84.20 EX: 5.96	S: 24.91 NS: 75.09 EX: 3.02	<0.0001	S: 19.15 NS: 80.85 EX: 3.81	S: 26.94 NS: 73.06 EX: 3.52	0.0001	S: 20.31 NS: 71.52 Ex: 8.17	S: 23.86 NS: 71.34 EX: 4.80	NS
Alcohol (%)	ND: 55.96 StD: 11.92 LD: 2.33	ND: 43.09 StD: 21.96 LD: 4.15	0.0021	ND: 46.36 StD: 19.15 LD: 3.58	ND: 45.51 StD: 20.15 LD: 3.88	NS	ND: 69,93 StD: 6.25 LD: 13.68	ND: 66,26 StD: 3.19 idem LD: 21.94	0.0085

Quantitative data are presented with mean and standard deviation and qualitative data as percentages

*BMI* Body mass index, *NC* Neck circumference, *CVCo* Cardiovascular Comorbidities, *ND* non drinkers, *StD* Strong drinkers (>3 unit/day), *LD* Lower drinkers

**Table 3:** Polysomnographic characteristics for all cardiometabolic comorbidities

	Diabetes *n* = 386	No diabetes *n* = 1331	*p* value	CVCo *n* = 671	No CVCo *n* = 1046	*p* value	Dyslipidemia *n* = 453	No dyslipidemia *n* = 1253	*p* value
AHI	45.82 ± 25.77	42.23 ± 25.60	0.0156	43.58 ± 24.59	42.50 ± 26.85	NS	44.12 ± 24.31	42.66 ± 26.18	NS
AHI/REM	50.01 ± 27.29	48.38 ± 26.99	NS	49.80 ± 26.77	47.61 ± 27.32	NS	47.53 ± 26.22	49.19 ± 27.34	NS
AHI/NREM	41.73 ± 26.26	38.46 ± 26.87	0.0342	39.40 ± 25.48	39.03 ± 28.13	NS	40.39 ± 25.19	38.79 ± 27.33	NS
SL (min)	70.01 ± 62.26	73.20 ± 62.06	NS	70.05 ± 59.99	75.20 ± 64.20	NS	68.20 ± 60.82	74.11 ± 62.48	NS
SE (%)	63.61 ± 15.05	67.42 ± 14.57	0.0001	64.89 ± 14.94	68.30 ± 14.45	0.0001	64.65 ± 14.65	67.21 ± 14.80	0.0022
TST (min)	327.12 ± 78.35	343.64 ± 76.60	0.0002	331.59 ± 79.02	348.56 ± 74.81	0.0001	330.79 ± 79.06	343.04 ± 76.64	0.0052
N1 (%)	4.55 ± 4.56	4.13 ± 3.62	NS	4.56 ± 4.27	3.90 ± 3.39	0.0156	4.92 ± 4.74	3.99 ± 3.48	<0.0001
N2 (%)	50.88 ± 16.37	50.36 ± 15.89	NS	50.61 ± 15.50	50.40 ± 16.50	NS	51.27 ± 15.81	50.22 ± 16.03	NS
N3 (%)	29.11 ± 17.19	28.67 ± 15.79	NS	28.80 ± 15.81	28.69 ± 16.41	NS	27.77 ± 16.28	29.10 ± 16.01	NS
REM (%)	15.52 ± 7.32	16.91 ± 6.88	0.0006	16.16 ± 7.29	17.04 ± 6.65	0.0091	16.17 ± 7.34	16.72 ± 7.34	NS
ARI	39.91 ± 18.34	37.13 ± 17.94	0.0007	37.91 ± 17.04	37.67 ± 19.14	NS	39.39 ± 17.55	37.20 ± 17.55	0.0356
Mean duration AH REM (sec)	25.59 ± 11.00	26.36 ± 9.69	NS	26.46 ± 10.41	25.89 ± 9.56	NS	27.09 ± 11.68	25.83 ± 11.68	0.0429
Mean duration AH NREM (sec)	21.90 ± 6.02	22.41 ± 5.96	NS	22.32 ± 5.73	22.24 ± 6.24	NS	22.63 ± 6.17	22.15 ± 6.17	NS
PLM	6.31 ± 22.47	5.63 ± 16.80	NS	6.23 ± 18.97	5.26 ± 17.33	NS	5.87 ± 19.18	5.73 ± 17.83	NS
ODI	57.42 ± 32.30	45.37 ± 30.60	<0.0001	50.44 ± 30.42	45.79 ± 32.36	0.0357	50.28 ± 31.87	47.60 ± 31.87	NS
Mean 02 saturation TST (%)	92.81 ± 30.94	92.18 ± 2.67	NS	92.27 ± 20.47	92.35 ± 2.75	NS	91.54 ± 2.88	92.59 ± 2.88	NS
02 desaturation < 90% (min)	102.33 ± 115.04	74.44 ± 99.13	<0.0001	91.67 ± 108.71	69.54 ± 97.24	<0.0001	96.06 ± 113.31	75.47 ± 113.31	0.0005
Minimal 02 saturation (%)	75.14 ± 10.42	77.58 ± 9.41	<0.0001	76.43 ± 9.64	77.62 ± 9.83	0.0203	76.20 ± 9.78	77.28 ± 9.78	NS
Mean HR (beats/min)	70.78 ± 10.36	68.10 ± 9.95	<0.0001	67.88 ± 10.25	69.63 ± 9.85	0.0003	68.20 ± 10.20	69.91 ± 10.20	NS

*AHI* Apnea-hypopnea index, *AH* Apnea-hypopnea, *REM* Rapid eye movement sleep, *NREM* Non-rapid eye movement sleep, *CVCo* Cardiovascular Comorbidities, *SL* Sleep latency, *TST* Total sleep time, *SE* Sleep efficiency, *N1* Sleep stage 1, *N2* Sleep stage 2, *N3* Sleep stage 3, *PLM* Periodic leg movements index, *ODI* Oxygen desaturation index, *ARI* Arousal index, *02* Oxygen, HR: heart rhythm. All the results are expressed as Mean + SD. NS: not significant

In multiple regression modeling, diabetes was significantly associated with female sex, increasing age, enlarged NC, high BMI, former or current smoking status, and less alcohol consumption. Severity of OSA (reflected by AHI), decreased sleep quality (reflected by low SE, TST, N3, REM), sleep fragmentation (ARI), nocturnal intermittent/global hypoxemia, PLM index and a higher mean nocturnal HR were predictors of diabetes. When adjusted for age, BMI, and smoking status, all of these predictors remained significant, and the duration of sleep AH in REM/NREM also became a determinant factor (Table 4).

**Table 4:** Linear regression analysis for the influence of diabetes-independent variables on quantitative dependent variables

		Model 1: diabetes			Model 2: diabetes + adjustement (age, BMI, smoking)		
	Dependent variables	R	R^2^	*P* value	R	R^2^	*P* value
Demographic data	NC	0.091	0.008	<0.0001	0.434	0.186	<0.0001
	Alcohol	0.089	0.007	0.002	0.224	0.047	<0.0001
Polysomnographic data	AHI	0.060	0.003	0.015	0.287	0.080	<0.0001
	AHI / REM	0.029	0.000	NS	0.345	0.117	<0.0001
	AHI / NREM	0.053	0.002	0.031	0.260	0.066	<0.0001
	SE (%)	0.111	0.012	<0.0001	0.204	0.039	0.0001
	TST (min)	0.093	0.008	<0.0001	0.189	0.033	<0.0001
	N3 (%)	0.006	−0.001	NS	0.124	0.013	<0.0001
	REM (%)	0.089	0.007	<0.0001	0.169	0.026	<0.0001
	ARI	0.068	0.001	0.005	0.179	0.030	<0.0001
	Mean duration AH REM (sec)	0.043	0.001	NS	0.202	0.038	<0.0001
	Mean duration AH NREM (sec)	0.044	0.001	NS	0.303	0.090	<0.0001
	ODI	0.167	0.027	<0.0001	0.364	0.128	<0.0001
	PLM	0.013	<0.0001	NS	0.141	0.018	<0.0001
	Mean 02 saturation TST (%)	0.019	0.000	NS	0.130	0.014	<0.0001
	02 desaturation < 90% (min)	0.109	0.011	<0.0001	0.341	0.114	<0.0001
	Minimal 02 saturation (%)	0.111	0.012	<0.0001	0.282	0.077	<0.0001
	Mean HR (beats/min)	0.108	0.011	<0.0001	0.373	0.137	<0.0001

*BMI* Body mass index, *NC* Neck circumference, *AHI* Apnea-hypopnea index, *AH* Apnea-hypopnea, *REM* Rapid eye movement sleep, *NREM* Non-rapid eye movement sleep, *SL* Sleep latency, *TST* Total sleep time, *SE* Sleep efficiency, *N3* Sleep stage 3, *PLM* Periodic leg movements index, *ODI* Oxygen desaturation index, *ARI* Arousal index, *HR* Heart rhythm,*0*_*2*_ Oxygen. All the results are expressed as Mean + SD. *NS* Not significant

In logistic regression, the results showed significant values between REM predominant OSA and diabetes status (Table 5).

**Table 5:** Logistic regression analysis for the influence of diabetes-independent variables on qualitative dependent variables, adjusted for age, BMI, and smoking status

	Variables	Odd ratio	95% confidence interval	*P* value
Demographic	Sex	0.891	0.688–1.155	NS
Polysomnography	Supine predominant OSA	0.990	0.683–1.153	NS
	REM predominant OSA	0.712	0.548–0.926	0.011

*REM* Rapid eye movement sleep, *OSA* Obstructive sleep apnea syndrome

### Cardiovascular comorbidities (CVCo)

Comparing patients with CVCo and without, CVCo patients were older, more often women, and non-smokers. They also exhibited worse sleep quality, more marked intermittent/global nocturnal hypoxemia, and a lower mean HR (Tables 2 and 3).

In multiple regression modeling, the presence of >1 CVCo was associated with enlarged NC and alcohol consumption, but not with age, sex, BMI, and smoking status. Severity of OSA, decreased sleep quality, PLM index and nocturnal intermittent/global hypoxemia were predictors of CVCo. When adjusted for age, BMI, dyslipidemia, and smoking status, all of these predictors remained significant (Table 6).

**Table 6:** Linear regression analysis for the influence of multiple cardiac-independent variables (CVCo) on quantitative dependent variables

		Model 1: CVCo			Model 2: CVCo + adjustement (age, BMI, smoking, sex, dyslipidemia)		
	Dependent variables	R	R^2^	*P* value	R	R^2^	*P* value
Demographic data	NC	0.092	0.007	0.001	0.722	0.519	<0.0001
	Alcool	0.095	0.007	0.005	0.263	0.065	<0.0001
Polysomnographic data	AHI	0.060	0.002	0.049	0.352	0.120	<0.0001
	AHI / REM	0.064	0.003	0.034	0.348	0.118	<0.0001
	AHI / NREM	0.054	0.002	NS	0.341	0.112	<0.0001
	SE (%)	0.064	0.003	0.047	0.268	0.067	<0.0001
	TST (min)	0.079	0.005	0.010	0.376	0.137	<0.0001
	N3 (%)	0.023	0.001	NS	0.074	0.001	NS
	REM (%)	0.130	0.016	<0.0001	0.215	0.042	<0.0001
	ARI	0.120	0.013	<0.0001	0.196	0.034	<0.0001
	Mean duration AH REM (sec)	0.092	0.007	0.001	0.200	0.036	<0.0001
	Mean duration AH NREM (sec)	0.021	0.001	NS	0.217	0.043	<0.0001
	PLM	0.027	< 0.0001	NS	0.142	0.018	<0.0001
	ODI (h)	0.009	0.001	NS	0.250	0.059	<0.0001
	Mean 02 saturation TST (%)	0.101	0.009	< 0.0001	0.176	0.027	<0.0001
	02 desaturation < 90% (min)	0.067	0.003	0.024	0.261	0.064	<0.0001
	Minimal 02 saturation (%)	0.092	0.007	0.001	0.283	0.076	<0.0001
	Mean HR (beats/min)	0.121	0.014	<0.0001	0.363	0.128	<0.0001

*CVCo* Cardiovascular Comorbidities, *BMI* Body mass index, *NC* Neck circumference, *AHI* Apnea-hypopnea index, *AH* Apnea-hypopnea, *REM* Rapid eye movement sleep, *NREM* Non-rapid eye movement sleep, *SL* Sleep latency, *TST* Total sleep time, *SE* Sleep efficiency, *N3* Sleep stage 3, *PLM* Periodic leg movements index, *ODI* Oxygen desaturation index, *ARI* Arousal index, *0*_*2*_ Oxygen, *HR* Heart rhythm. All the results are expressed as Mean + SD. *NS* Not significant

When we evaluated qualitative dependent variables, the logistic regression resulted in non-significant results between CVCo and supine or REM predominant OSA.

### Dyslipidemia

Comparing dyslipidemic and non-dyslipidemic patients, dyslipidemic patients were older, had a larger NC, and were more often drinkers. They also exhibited worse sleep quality and longer AH in REM sleep (Tables 2 and 5).

In multiple regression modeling, the presence of dyslipidemia was associated with enlarged NC, low REM percentage, and longer AH in REM sleep. When adjusted for age, sex, and BMI, dyslipidemia was associated with the preceding factors and the absence of alcohol consumption, OSA severity, decreased sleep quality, PLM index and lower nocturnal HR. (Table 7).

**Table 7:** Linear regression analysis for the influence of dyslipidemia-independent variables on quantitative dependent variables

		Model 1: Dyslipidemia			Model 2: Diyslipidemia + adjustement (age, BMI, sex)		
	Dependent variables	R	R^2^	*P* value	R	R^2^	*P* value
Demographic data	NC	0.058	0.003	0.018	0.710	0.502	<0.0001
	Alcool	0.032	0.000	NS	0.190	0.033	<0.0001
Polysomnographic data	AHI	0.023	0.000	NS	0.351	0.121	<0.0001
	AHI / REM	0.027	0.000	NS	0.353	0.123	<0.0001
	AHI / NREM	0.023	0.000	NS	0.338	0.112	<0.0001
	SE (%)	0.036	0.001	NS	0.251	0.061	<0.0001
	TST (min)	0.018	0.000	NS	0.378	0.141	<0.0001
	N3 (%)	0.043	0.001	NS	0.068	0.002	NS
	REM (%)	0.074	0.005	0.002	0.204	0.039	<0.0001
	ARI	0.067	0.004	0.005	0.182	0.031	<0.0001
	Mean duration AH REM (sec)	0.104	0.010	<0.0001	0.182	0.031	<0.0001
	Mean duration AH NREM (sec)	0.027	0.000	NS	0.200	0.038	<0.0001
	PLM	0.001	0.001	NS	0.139	0.017	<0.0001
	ODI	0.035	0.001	NS	0.229	0.050	<0.0001
	Mean 02 saturation TST (%)	0.033	0.000	NS	0.153	0.024	<0.0001
	02 desaturation < 90% (min)	0.051	0.002	0.036	0.252	0.061	<0.0001
	Minimal 02 saturation (%)	0.054	0.002	0.030	0.277	0.074	<0.0001
	Mean HR (beats/min)	0.035	0.001	NS	0.353	0.123	<0.0001

BMI: body mass index, NC: neck circumference, AHI: apnea-hypopnea index, AH: apnea-hypopnea, REM: rapid eye movement sleep, NREM: non-rapid eye movement sleep, SL: sleep latency, TST: total sleep time, SE: sleep efficiency, N3: sleep stage 3, PLM: periodic leg movements index, ODI: oxygen desaturation index, ARI: arousal index, 0_2_: oxygen, HR: heart rhythm. All the results are expressed as Mean + SD. NS: not significant

In logistic regression analysis only REM predominant OSA was associated with dyslipidemia (OR: 0.742 _[0.579–0.950]_, *p* = 0.018).

## Discussion

In this large retrospective study, we have shown that in moderate-to-severe OSA, the presence of cardiometabolic complications is associated with a large neck and a particular polysomnographic pattern for diabetes, dyslipidemia, and cardiovascular disease. Indeed, correlations between polysomnographic parameters and the presence of cardiometabolic complications, after adjustment for confounding factors, show that AHI, AH duration, reduced sleep efficiency and total sleep time, low REM proportion, and nocturnal hypoxemia are risk factors associated with all complications. Moreover, absence of alcohol consumption is also associated with metabolic disorders.

OSA is associated with an increased risk of metabolic syndrome [12], including dyslipidemia and insulin resistance, and multiple cardiovascular comorbidities, such as hypertension, coronary artery disease, arrhythmias, ischemic stroke, and congestive heart failure [13, 14]. Is it surprising that all of these comorbidities share the same polysomnographic picture?

Severity of OSA is typically described by the AHI, measuring the number of obstructive events during each hour of sleep. Occurrence of comorbidities increases in parallel with OSA severity.

Indeed, nearly 20 years ago, a linear relationship between AHI and incidental HTA was described by Peppard et al. [15], in a cohort of 709 patients followed for 4 years [15]. Similarly, the risk of stroke or death has been shown to be correlated with OSA severity in a large series of 1022 OSA patients who were followed for 3.4 years [16]. Oxidative stress, endothelial dysfunction, systemic inflammation, and activation of the sympathetic nervous system are the pathophysiological mechanisms leading to cardiovascular disorders in OSA, as a consequence of repeated obstructive sleep apnea and hypopnea, inducing hypoxia/hypercapnia, arousals, and negative intra-thoracic pressure [17].

Ip et al. have shown that insulin resistance is independently associated with increasing AHI [18]. In untreated diabetic patients, HbA1c levels have been shown to be positively associated with AHI [19]. Recent data from the European ESADA cohort have shown that the prevalence of diabetes increases with OSA severity, reaching 29% in patients with severe OSA [20]. Underlying mechanisms include intermittent hypoxia and sleep fragmentation, activation of the sympathetic nervous system, oxidative stress, systemic inflammation, alterations in appetite-regulating hormones, and activation of the hypothalamic-pituitary-adrenal axis which leads first to the development of insulin resistance and then progresses to glucose intolerance and diabetes [21]. Guan et al., in an observational cohort, have also shown a linear dose effect between AHI and dyslipidemia [22]. Altogether, our results confirm this strong association between OSA severity, reflected by AHI, with the presence of cardiometabolic comorbidities that share the same pathophysiological mechanisms.

However, in clinical practice, we know that the widely used AHI is not sufficiently accurate to describe the full picture of OSA. In the present study, we have highlighted that mean duration of AH in REM and non-REM sleep is also an independent predictor of the presence of cardiometabolic comorbidities. Wu et al., have shown, in a retrospective study, that a longer mean AH duration (but not AHI) is associated with higher odds of moderate-to-severe HTA [23]. Therefore, clinicians should be advised to pay attention not only to AHI but also to HA length in order to assess OSA severity.

The same group has shown, in a rat model, that longer AH duration is associated with more systemic inflammatory and endothelial dysfunction, that can (partially) recover when duration decreases or when sleep apneas are suppressed [24].

It is interesting to note that heart rate excursion after an obstructive apnea is associated with apnea duration and not with AHI [25]. Longer apnea duration also implies deeper desaturation, which has been shown to be associated with a higher risk of atrial fibrillation [26].

Future prospective studies are needed to discern the specific impact of AHI and of the mean duration of AH on comorbidities and symptoms.

We have also to stress that if one wants to use AH duration as a metric in the future, it is mandatory to rely on manually scored AH according to AASM scoring rules [9] and not to automated measurements made by the PSG systems, because the AH detection algorithms can vary from one device to another.In this study, poor sleep was also associated with the presence of cardiometabolic comorbidities.

Independently from OSA, poor sleep (increased sleep latency, wake after sleep onset, reduced SE, TST, N3, and REM sleep) have been demonstrated as risk factors for diabetes [27, 28], with 13–21% of insomniacs affected by the disorder. Patients suffering from insomnia and diabetes have more comorbidities (e.g. HTA, dyslipidemia), are generally overweight/obese, and have a short sleep duration, from less than 390 min [28]. Insomnia could also help identifying more severe patients. Indeed, it has been shown, in elderly community-dwelling adults, that comorbid insomnia was related to hypoxemia in OSA patients suffering from cardiovascular disease, and associated to a worse prognosis [29].

In our study, polysomnographic markers of decreased sleep quality (reduced SE, TST, N3, and REM sleep, and increased ARI) were correlated with the presence of cardiometabolic comorbidities, and may be a consequence of OSA or an associated primary insomnia, which occurs frequently in OSA, in about 44–55% of patients [30]. This comorbid insomnia has a negative additive effect on comorbidities through the activation of the hypothalamic-pituitary-adrenal axis and excessive sympathetic nervous activity [28].

Sleep fragmentation, a typical pattern of OSA, is associated with decreased insulin sensitivity in healthy subjects, through increases in sympathetic nervous system and adrenocortical activity [31] and can aggravate the metabolic effects of short sleep duration in OSA patients.

Short sleep duration, less than 6 h/night, was also shown to be associated with diabetes incidence but not with incidental cardiovascular disease in a very large meta-analysis [32]. Underlying pathophysiological mechanisms include decreased glucose tolerance and insulin sensitivity, increased evening concentrations of cortisol and levels of ghrelin, decreased levels of leptin, and increased hunger and appetite [33]. Prolonged short sleep durations could also lead to hypertension through raised 24-h blood pressure and increased salt retention [34].

PLM severity was also associated with cardiometabolic disorders. It has been documented that PLM induces blood pressure elevations and heart rate elevations [35]. These two markers are now recognized as surrogates for cardiovascular risk, such that our findings are not surprising. Yet, no data have been published regarding the association of PLM and metabolic disorders. There are however published data regarding the impact of sleep fragmentation on glucose metabolism disorders in humans [21] and on dyslipidemia in mice [36]. As PLM induce sleep fragmentation [37, 38], it seems to be the most plausible underlying physiopathological mechanisms.

Finally, nocturnal hypoxemia was invariably correlated with the presence of cardiometabolic comorbidities.

Intermittent hypoxia is the hallmark of OSA. It is characterized by repetitive profound hypoxic periods followed by blood reoxygenation. It has been hypothesized that these phenomena are similar to repeated ischemia and reperfusion events which result in release of reactive oxygen species and activate oxidative stress pathways, promoting cardiovascular morbidity in OSA [39].

OSA seems also to promote post-prandial hyperlipidemia through intermittent hypoxia. It has been demonstrated first in a mouse model [40], then in humans by Phillips et al. [41], showing that in humans with OSA, post-prandial lipids are elevated and favorably impacted by CPAP [41]. The benefit of CPAP on total cholesterol levels was confirmed in a recent meta-analysis [42].

Intermittent hypoxia seems also to play a role in glucose metabolism disorders. Indeed, Tassone et al. have compared obese and OSA obese patients and have shown that OSA obese patients exhibit more insulin resistance, independently of fat distribution, suggesting a role of intermittent hypoxia [43].

In our retrospective series, patients suffering from cardiometabolic comorbidities also had a larger NC than their healthier OSA counterparts. Even after adjustment for BMI, enlarged NC remained a risk factor. Fat accumulation in the neck is a sign of visceral obesity, responsible for OSA and the metabolic syndrome [12], and an additional factor promoting cardiovascular comorbidities [44].

Our last finding regards alcohol consumption. There were more metabolic comorbidities in non-drinkers and more cardiovascular disorders in drinkers. Recent data on the metabolic syndrome have confirmed a lower prevalence in patients who eat seafood and non-starchy vegetables, and drink moderate amounts of alcohol [45], in concordance with our findings. Moderate alcohol consumption has been shown to increase insulin sensitivity [46], and to contributes to a lower risk of diabetes development in Japanese men [47]. It leads also to lower HDL-c levels and adiponectin, which inhibits liver fat accumulation [48]. Regarding cardiovascular disorders, the negative effect of alcohol is dose-dependent and related to the type of ingested alcohol [49, 50], and it is thus not surprising to see a negative effect of alcohol in our population.

### Limitations

The results of this study come from retrospective data analysis. This design could potentially lead to missing data or biases. Moreover, in the absence of control group (patients without OSA), it is difficult to ascertain the associations between sleep parameters and cardiometabolic comorbidities. Thus, our results should be confirmed in prospective/controlled series.

## Conclusions

This study identifies particular demographic and polysomnographic factors associated with cardiometabolic comorbidities. Indeed, patients (especially women) suffering from more severe OSA, longer sleep apneas and hypopneas, worse sleep quality, and marked intermittent/global nocturnal hypoxemia are more likely to develop cardiometabolic comorbidities. Particular attention should be paid to all these parameters when choosing OSA treatment on an individual basis. This should also stimulate clinicians to obtain and maintain adequate treatment in this population, in order to prevent future complications. Moreover, further studies need to be conducted, prospectively, to identify the added value of comorbid insomnia in the occurrence of cardiometabolic comorbidities in OSA.

## Data Availability

The datasets used and/or analysed during the current study are available from the corresponding author on reasonable request.

## Abbreviations

02: Oxygen
AH: Apnea- hypopnea
AHI: Apnea- hypopnea index
ARI: Arousal index
BMI: Body Mass Index
CPAP: Continuous positive airway pressure
CVCo: Cardiovascular comorbidity
HR: Heart rhythm
HTA: Hypertension
N1: sleep stage 1
N2: sleep stage 2
N3: sleep stage 3
NC: Neck Circumference
NREM: Non- Rapid Eye Movement
ODI: Oxygen Desaturation Index
OSA: Obstructive Sleep Apnea Syndrome
PLM: Periodic Leg Movements
PSG: Polysomnography
REM: Rapid Eye Movement
SE: Sleep Efficiency
TST: Total Sleep Time
